# Transcriptomic analysis of *OsRUS1* overexpression rice lines with rapid and dynamic leaf rolling morphology

**DOI:** 10.1038/s41598-022-10784-x

**Published:** 2022-04-25

**Authors:** Ning Yu, Yaping Liang, Qingping Wang, Xinxiang Peng, Zhenghui He, Xuewen Hou

**Affiliations:** 1 Center for Photosynthesis and Plant Stress Biology, College of Life Sciences, South-China Agricultural University, Guangzhou, 510642 China; 2 State Key Laboratory for Conservation and Utilization of Subtropical Agro-Bioresources, College of Life Sciences, South China Agricultural University, Guangzhou, 510642 China; 3 Department of Biology, San Francisco State University, 1600 Holloway Avenue, San Francisco, CA 94132 USA

**Keywords:** Molecular biology, Plant sciences

## Abstract

Moderate leaf rolling helps to form the ideotype of rice. In this study, six independent *OsRUS1-GFP* overexpression (*OsRUS1-OX*) transgenic rice lines with rapid and dynamic leaf rolling phenotype in response to sunlight were constructed. However, the mechanism is unknown. Here, RNA-Seq approach was utilized to identify differentially expressed genes between flag leaves of *OsRUS1-OX* and wildtype under sunlight. 2920 genes were differentially expressed between *OsRUS1-OX* and WT, of which 1660 upregulated and 1260 downregulated. Six of the 16 genes in GO: 0009415 (response to water stimulus) were significantly upregulated in *OsRUS1-OX*. The differentially expressed genes between WT and *OsRUS1-OX* were assigned to 110 KEGG pathways. 42 of the 222 genes in KEGG pathway dosa04075 (Plant hormone signal transduction) were differentially expressed between WT and *OsRUS1-OX*. The identified genes in GO:0009415 and KEGG pathway dosa04075 were good candidates to explain the leaf rolling phenotype of *OsRUS1-OX*. The expression patterns of the 15 genes identified by RNA-Seq were verified by qRT-PCR. Based on transcriptomic and qRT-PCR analysis, a mechanism for the leaf rolling phenotype of *OsRUS1-OX* was proposed. The differential expression profiles between WT and *OsRUS1-OX* established by this study provide important insights into the molecular mechanism behind the leaf rolling phenotype of *OsRUS1-OX*.

## Introduction

Rice is a staple food for humans, especially for people in Asia. Due to the decrease of arable land and the increase of human population, the demand for breeding higher-productivity rice cultivars is increasing. Rice ideotype is one of the important indicators for higher-productivity rice cultivar breeding. The shape of the leaf is considered an important agronomic trait and has been associated with the super-rice ideotype^1^. Additionally, moderate leaf rolling is an important characteristic for the super-rice ideotype^2^. Due to better light transmission rate and absorption efficiency, moderate leaf rolling can help to improve photosynthetic efficiency and grain yields^3–5^. Therefore, isolating leaf rolling genes should be beneficial for breeding rice cultivars with ideotype.

Although the rice leaf rolling phenotype is relatively simple, the mechanisms behind it are various and complex. There are two types of rice leaf rolling: rice leaf developmental-gene-mutation related; and environmental factors induced. The formation of a rice leaf is a complex process, which includes the development of phyllopodium, the adaxial-abaxial axis, bulliform cells, sclerenchymatous cells, and the cuticle, along with the regulation of osmotic pressure or turgidity in bulliform cells^6–9^. The mutation of genes in these processes will cause abnormal development in rice leaves, including leaf rolling. Currently, more than fourteen *rolling leaf* (*rl*) mutants in rice have been isolated; however, only *RL9* and *RL14* have been cloned and studied in detail. *SHALLOT-LIKE1* (*SLL1*), a gene involved in the regulation of leaf rolling through the regulation of sclerenchyma cell differentiation, encodes a MYB transcription factor of the KANADI family^10,11^. *SEMI-ROLLEDLEAF1* (*SRL1*), a gene involved in the regulation of leaf rolling through the inhibition of bulliform cell formation, encodes a putative glycosylphosphatidyl inositol-anchored protein^5^. The *SEMI-ROLLED LEAF2* (*SRL2*) gene encodes a novel plant-specific protein, which functions in the regulation of rice leaf rolling, and may be involved in the trans-differentiation process from mesophyll cells to sclerenchymatous cells^12^. The second type of rice leaf rolling is induced by environmental factors, such as temperature, humidity and light, and is also called the environment inducible phenotype^13^. The leaves of *rl15(t)* become highly rolled at cloudless noon, but are expanded at other times^14^. *constitutively wilted 1* (*oscow1*), a T-DNA insertion mutant of rice, has leaf rolling that is significantly reduced under low light and high relative humidity. It was postulated that *oscow1* mutants may be light or/and humidity inducible. OsCOW1, a YUCCA family protein, is encoded by *Os03g06654*, and is involved in the biosynthesis of IAA^15^. The leaves of the rice CM2088 mutant roll markedly under well-watered sunny conditions, but the rolling of its leaves was not evident during early morning and evening^16^. To date, there are only a small number of environment-induced leaf-rolling mutants identified in rice.

*RUS* (*ROOT UV-B SENSITIVE*) genes were firstly identified in *Arabidopsis*. *AtRUS1*/*WXR3* (*WEAK AUXIN RESPONSE 3*) and *AtRUS2*/*WXR1* have functions involved in very-low fluence (VLF) UVB responses and vitamin B6 (VB6) homeostasis^17–19^, or regulation of polar auxin transport^20,21^. Recently, *Arabidopsis RUS4* was reported to play a role in male fertility^22^, and *RUS6* in early embryo development^23^. However, no leaf rolling phenotype of *AtRUS*/*WXR* genes has been reported in *Arabidopsis*. There are six *OsRUS* genes in the rice genome; however, their functions are largely unknown^24^. *OsRUS1* (*Os04g22360*) is one of the six *OsRUS* genes. OsRUS1 was predicted to localize in chloroplast by available bioinformatics tools, and further confirmed by transient expression of OsRUS1(1-160aa)-GFP in rice protoplast^24^. In this study, six independent *OsRUS1-GFP* overexpression transgenic rice lines (here after *OsRUS1-OX*) and empty vector control lines were constructed using ZH11 (here after WT) as the background. The leaves of *OsRUS1-OX* lines were significantly rolled under sunlight, while their leaves were expanded in the morning, evening, night, and shaded daytime. Furthermore, the rolled leaves of *OsRUS1-OX* lines expanded in about seven minutes when the sunlight was shaded by cloud or paperboard. The expanded leaves rolled again in about four minutes after the paperboard was taken away. While the empty vector control lines did not have this phenotype, and show no difference with WT. To the best of our knowledge, this is the first report of a fast-speed environmentally-induced rice leaf rolling/expanding phenotype. In order to understand the mechanism of this phenotype, an RNA-Seq approach combined with digital gene expression profile (DGE) analysis was used to rapidly identify and analyze the differentially expressed genes between WT and the *OsRUS1-OX* lines. These results provided insights into the transcriptional levels of key genes in the process of leaf rolling in the *OsRUS1-OX* lines. Additionally, analysis of the functional pathways of these genes provided a better understanding of the mechanism of the rapid and dynamic rice leaf rolling phenotype caused by *OsRUS1* overexpression.

## Results

### The rapid and dynamic leaf rolling and expanding of *OsRUS1-OX* lines

**Figure 1:**
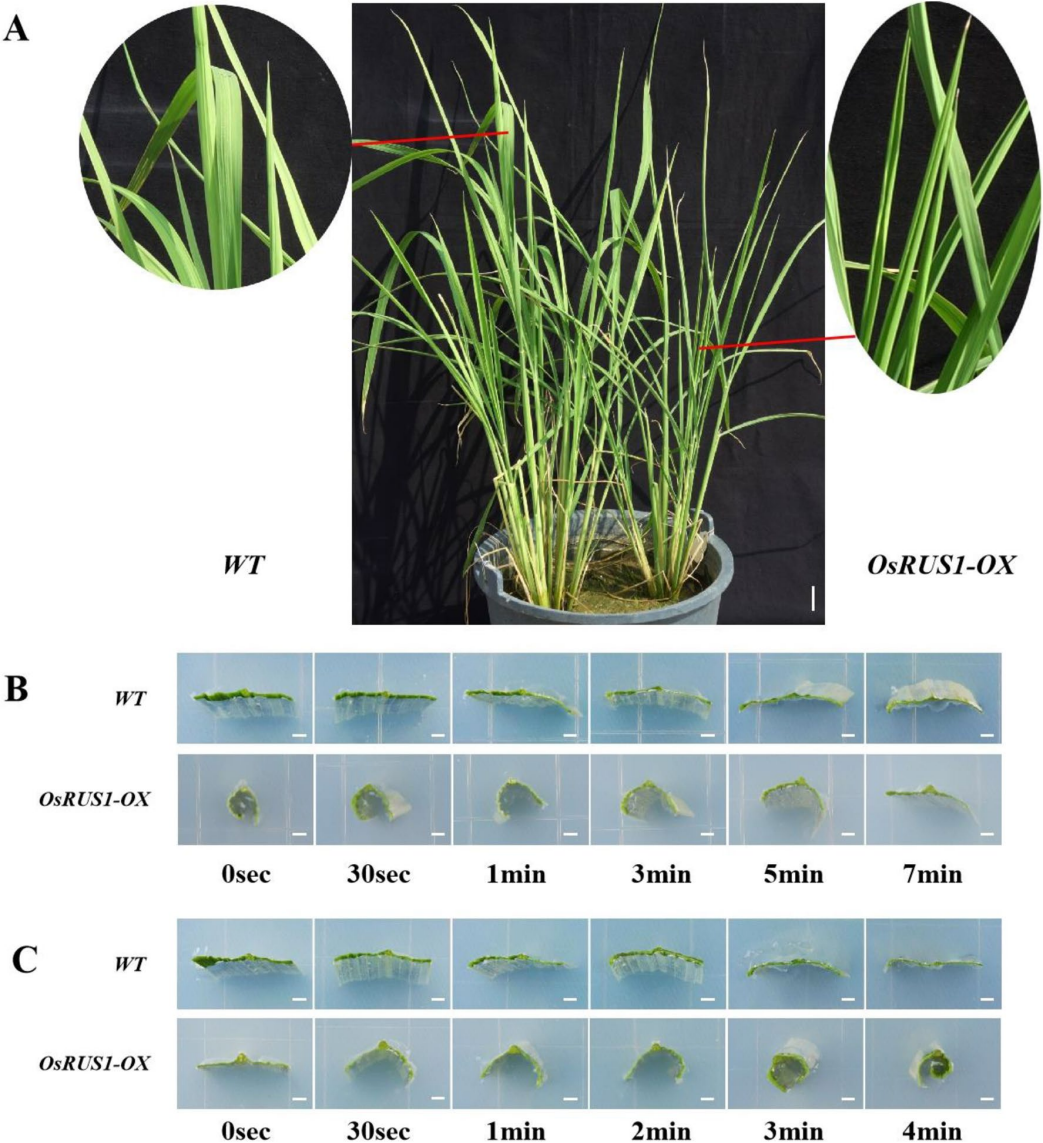
Leaf rolling morphology of WT and *OsRUS1-OX* in response to sunlight. (**A**) Morphology of the WT and *OsRUS1-OX* under sunlight, scale bar 2 cm; (**B**) The rapid leaf expanding in *OsRUS1-OX* when the sunlight was shaded, scale bar 2 mm; (**C**) The rapid leaf rolling in *OsRUS1-OX* in response to sunlight, scale bar 2 mm. WT, the wildtype of ZhongHua11; *OsRUS1-OX*, the *OsRUS1-GFP* Overexpression transgenic line.

In this study, at least six independent *OsRUS1-OX* lines were created, and all displayed a similar leaf rolling phenotype. One of the lines was chosen for further study here. The leaves of *OsRUS1-OX* were significantly rolled under sunlight, while the leaves of WT were fully expanded (Fig. 1A). When the sunlight was shaded by paperboard, the rolled leaves of *OsRUS1-OX* expanded in about seven minutes (Fig. 1B). When the paperboard was taken away, the expanded leaves of *OsRUS1-OX* rolled again in about four minutes (Fig. 1C).

### Transcriptional study of WT and *OsRUS1-OX* by RNA-Seq approach

In order to explore the mechanisms behind the leaf rolling phenotype, an RNA-Seq approach was used to study the transcriptional profiles of WT and *OsRUS1-OX*. RNA was extracted from the flag leaves of WT and *OsRUS1-OX* plants under sunlight, and sequenced on the Illumina Hiseq 2000 platform. This high-throughput RNA-Seq sequencing generated 24,995,822 to 38,693,740 raw sequencing reads for each sample. After removing adaptor sequences, ambiguous reads and low-quality reads, 24,376,218 to 37,815,434 high quality clean reads for each of the six samples remained. About 90% of the clean read data had Phred quality scores at the Q30 level (Table 1). The classification of raw reads, the error rate distribution, and the GC content distribution of the data are shown in Supplementary Figure S1, S2 and S3, respectively. These results confirmed that the quality of the six RNA-Seq data was excellent, and would provide reliable data for further data-mining.

**Table 1:** Summary of sequence assembly after Illumina sequencing.

Sample name	Raw reads	Clean reads	Clean bases	Error rate (%)	Q20 (%)	Q30 (%)	GC content (%)
WT1	24,995,822	24,376,218	3.05G	0.02	95.96	90.6	53.81
WT2	32,829,856	31,888,256	3.99G	0.02	96.67	92.06	53.2
WT3	31,913,804	31,093,862	3.89G	0.02	95.92	90.5	53.36
*OsRUS1-OX*_1	38,313,408	37,297,918	4.66G	0.02	96.65	92.05	54.93
*OsRUS1-OX*_2	34,353,870	33,516,652	4.19G	0.02	96.45	91.59	54.9
*OsRUS1-OX*_3	38,693,740	37,815,434	4.73G	0.02	96.76	92.19	55.18

Sample name, the name of samples; Raw reads, the original sequence data; Clean reads, filtered Raw reads, (subsequent biological information analysis was based on Clean reads); Clean bases, the number of Clean reads was multiplied by the length, and converted to G as the unit; Error rate, calculated by the formula: Qphred = − 10log_10_ (E); Q20 and Q30,The percentage of bases for which the Phred value was larger than 20 and 30, respectively; GC content, the sum of the number of bases G and C was calculated as a percentage of the total base number. WT, the wildtype of ZhongHua11; *OsRUS1-OX*, the *OsRUS1-GFP* Overexpression transgenic line.

The clean reads of all samples were mapped to the rice genome using TopHat^25^. About 84.83% to 88.7% of the total reads from the RNA-seq data of all the samples was mapped uniquely to the genome, while a small proportion of reads (1.17–2.13%) was mapped multiple times to the genome (Supplementary Table S2). The total reads of each sample were aligned to different regions of the rice reference genome, and the distribution ratio of reads that mapped to exons, introns and intergenic regions was analyzed. In the three WT libraries and the three *OsRUS1-OX* libraries, the number of reads that mapped to exons was greater than 90% (Supplementary Figure S4). The distribution and density of total mapped reads to rice chromosomes was also determined (Supplementary Figure S4). The number of reads that mapped to each chromosome, relative to chromosome length, showed that the reads were evenly distributed to each chromosome of rice (Supplementary Figure S5).

### Pearson correlation analysis between RNA-Seq samples

**Figure 2:**
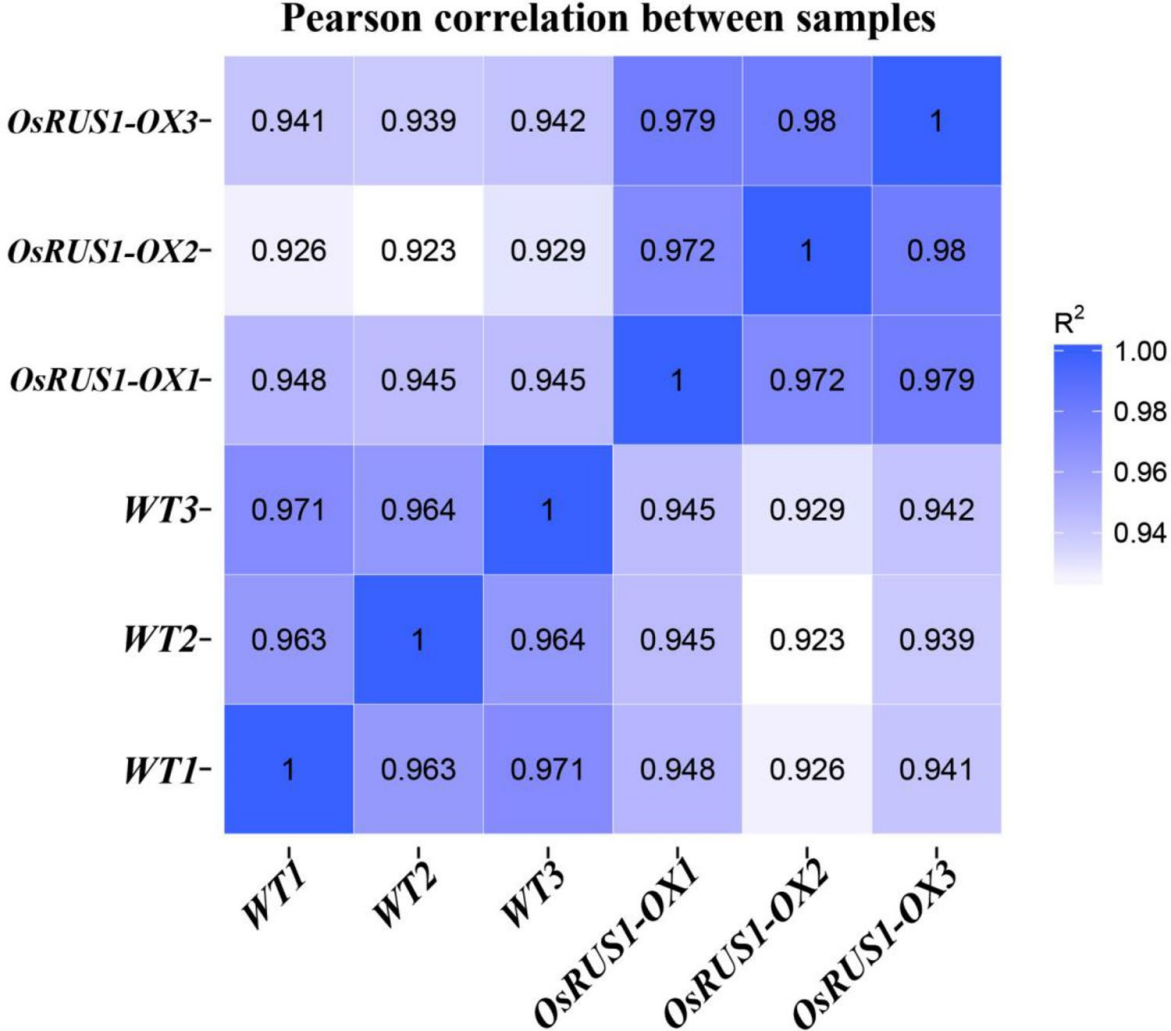
Pearson correlation between WT and *OsRUS1-OX* RNA-Seq data.The change of the square (R^2^) value of the correlation coefficient of Pearson is indicated by the change of the blue color. The deeper color indicates a bigger R^2^ value and a higher correlation between samples. WT, the wildtype of ZhongHua11; *OsRUS1-OX*, the *OsRUS1-GFP* Overexpression transgenic plant.

Correlation of gene expression levels between samples is an important indicator for results reliability and sample selection reasonability. The more the correlation coefficient approximates 1, the higher the similarity of expression patterns between samples is. The ENCODE (Encyclopedia of DNA Elements) scheme suggests that, under ideal sampling and experimental conditions, the square (R^2^) of the Pearson correlation coefficient should be greater than 0.92^26,27^. The R^2^ values between WT and *OsRUS1-OX* samples were greater than 0.92 and less than 0.95, and also less than that of the three WT samples and the three *OsRUS1-OX* samples, respectively. This indicated that there were differences in expression levels between WT and *OsRUS1-OX* (Fig. 2). The R^2^ values between the three WT samples were very close, which showed that the expression levels in those samples were similar. The same was true between the three *OsRUS1-OX* samples (Fig. 2). This indicated that our RNA-Seq analysis assay was reliable and that the selected samples were reasonable.

### The general gene expression profiles of WT and *OsRUS1-OX*

The most direct assessment of gene expression level is transcript abundance: the higher the transcript abundance, the higher the level of gene expression. In our RNA-Seq analysis, we estimated the level of gene expression by mapping the sequence reads to a genome region or a gene exon region. The count of reads should be proportional to the true gene expression level, but is also influenced by the length of the gene and sequencing depth. Therefore, Fragments Per Kilobase of transcript sequence per Million base pairs sequenced (FPKM) values are more reliable than simple read counts for the comparison of gene expression levels between different samples^28^. Generally speaking, an FPKM value less than 3 is considered to be low expression, 3 to 15 as medium expression, and more than 15 as high expression. All of the uniquely mapped reads were used for calculating the FPKM values (Table 2). Compared to WT, the number of low expression genes slightly decreased in *OsRUS1-OX* (WT 82.99%, *OsRUS1-OX* 82.54%), while the number of high expression genes slightly increased in *OsRUS1-OX* (WT 8.73%, *OsRUS1-OX* 9.16%) (Table 2). According to the FPKM distribution and FPKM density distribution data between WT and *OsRUS1-OX*, only a small number of genes had altered expression patterns (Supplementary Figure S6).

**Table 2:** Statistics of genes in different expression-level interval.

FPKM interval	WT1	WT2	WT3	*OsRUS1-OX*_1	*OsRUS1-OX*_2	*OsRUS1-OX*_3
0–1	71,691 (78.63%)	71,912 (78.87%)	71,524 (78.44%)	71,078 (77.95%)	71,436 (78.35%)	71,278 (78.17%)
1–3	3964 (4.35%)	3939 (4.32%)	3976 (4.36%)	4051 (4.44%)	3954 (4.34%)	3977 (4.36%)
3–15	7675 (8.42%)	7491 (8.22%)	7478 (8.20%)	7758 (8.51%)	7419 (8.14%)	7547 (8.28%)
15–60	5846 (6.41%)	5683 (6.23%)	5998 (6.58%)	6105 (6.70%)	6137 (6.73%)	6196 (6.80%)
> 60	2004 (2.20%)	2155 (2.36%)	2204 (2.42%)	2188 (2.40%)	2234 (2.45%)	2182 (2.39%)

FPKM, expected number of Fragments Per Kilobase of transcript sequence per Million base pairs sequenced. WT, the wildtype of ZhongHua11; *OsRUS1-OX*, the *OsRUS1-GFP* Overexpression transgenic line.

### The differentially expressed genes identified between WT and *OsRUS1-OX*

**Figure 3:**
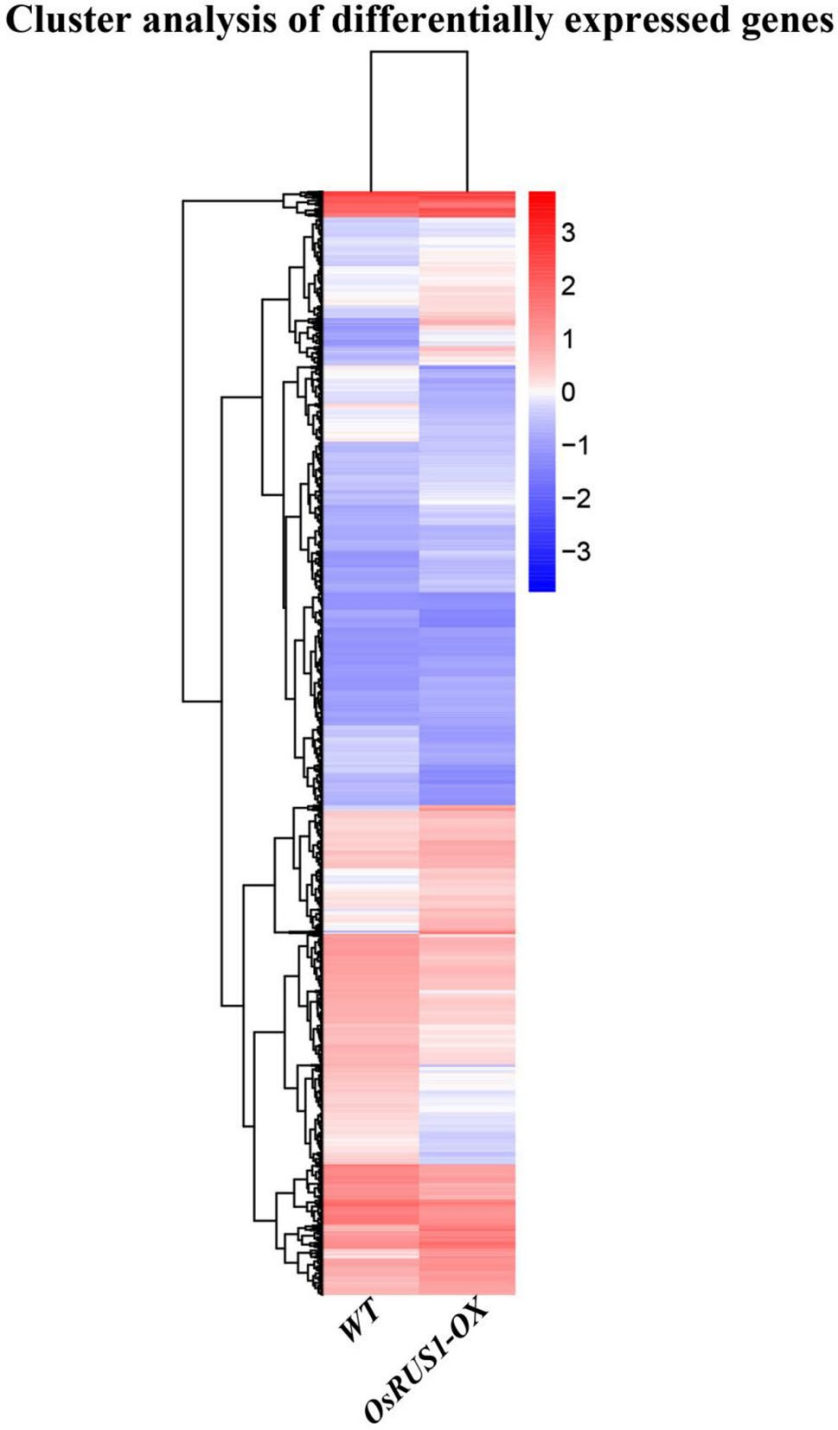
Hierarchical clustering of the differentially expressed genes between WT and OsRUS1-OX. The RNA-seq data derived from WT and *OsRUS1-OX* based on log_10_(FPKM + 1) values were utilized. The blue bands represent down regulated genes, and the red bands represent up regulated genes. WT, the wildtype of ZhongHua11; *OsRUS1-OX*, the *OsRUS1-GFP* Overexpression line.

The molecular basis of the dynamic leaf rolling phenotype of *OsRUS1-OX* may be facilitated by the genes that are differentially expressed between WT and *OsRUS1-OX*. In our experiments, there were a total of 2920 differentially expressed genes identified, of which 1660 were upregulated and 1260 were downregulated (Supplementary Table S3). A hierarchical clustering of the differentially expressed genes was made according to the log_10_(FPKM + 1) values of the two samples (Fig. 3). In this analysis, we observed that the number and fold change level of upregulated genes exceeded that of downregulated genes.

**Figure 4:**
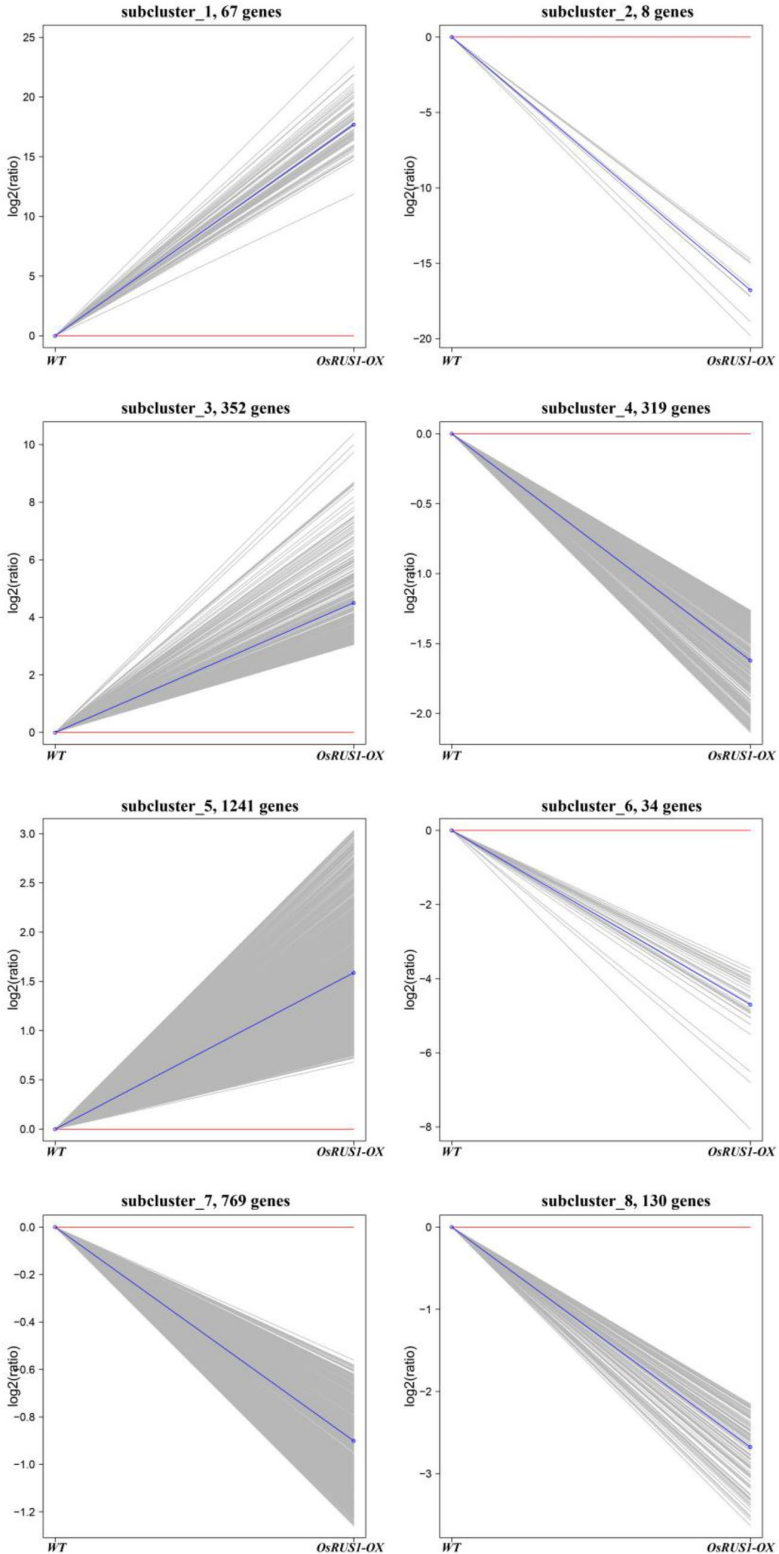
The k-means clustering of differentially expressed genes between WT and *OsRUS1-OX*. The eight major clusters obtained by K-means algorithm, representing upregulated (1, 3, 5), and downregulated (2, 4, 6, 7, 8) clusters. Expression ratios are expressed as Log_2_. WT, the wildtype of ZhongHua11; *OsRUS1-OX*, the *OsRUS1-GFP* Overexpression transgenic line.

The relative expression levels (log_2_(ratios)) of the differentially expressed genes were analyzed by a cluster analysis based on the K-means method^29^. Eight expression clusters of differentially expressed genes were identified (Fig. 4). The most abundant group was subcluster 5, with 1241 genes that showed a positive slope and were expressed at the highest levels in *OsRUS1-OX*. The upregulation amplitudes of the relative expression levels of genes in subcluster 1 were larger than those in subcluster 3 and subcluster 5. The second most abundant group was subcluster 7, with 769 genes that showed a negative slope and were downregulated in *OsRUS1-OX*. Subcluster 2, subcluster 4, subcluster 6 and subcluster 8 showed a similar pattern with subcluster 7, but the downregulated amplitudes of the relative expression levels of genes in subcluster 2 were the largest.

1385 genes were expressed only in *OsRUS1-OX*, and 1029 genes were expressed only in WT. Although there were more genes (18,551) expressed in both samples, only some of them were quantitatively regulated at different levels (see Supplementary Table S4 for the gene list for each category). From the patterns of gene expression, we predicted that the upregulation and downregulation of some genes might be the cause of the rapid and dynamic leaf rolling phenotype of *OsRUS1-OX* in response to sunlight.

**Figure 5:**
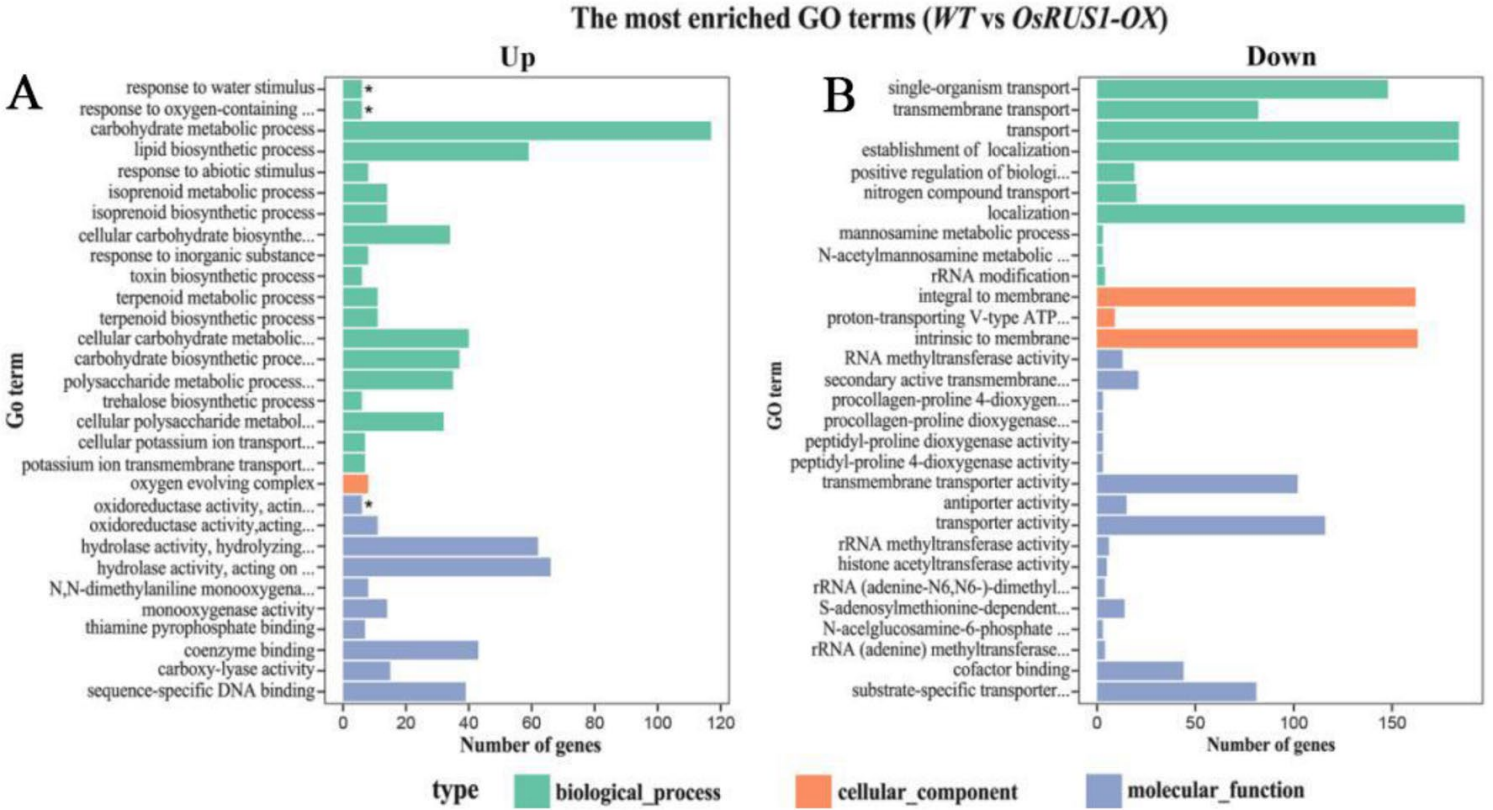
Histogram of Gene Ontology (GO) classification of differential expressed genes between WT and *OsRUS1-OX*. The results are summarized in three main categories: biological process (BP), cellular component (CC), and molecular function (MF), shown in green, red, and blue, respectively. The Y-axis indicates enriched Gene Ontology (GO) terms; the X-axis indicates the number of differential genes in a category; *, significantly enriched GO term. A. significantly enriched Gene Ontology (GO) terms in the upregulated genes in the leaves of *OsRUS1-OX* as compared to WT; B. significantly enriched Gene Ontology (GO) terms in the downregulated genes in the leaves of *OsRUS1-OX* as compared to WT *OsRUS1-OX*. WT, the wildtype of ZhongHua11; *OsRUS1-OX*, the *OsRUS1-GFP* Overexpression transgenic line.

In order to clarify their functions, genes that were significantly differentially expressed between WT and *OsRUS1-OX* were classified into different functional categories using GOseq^30^. The annotations were verified and integrated using gene ontology (GO) classification (http://www.geneontology.org/). All of the significantly differentially expressed genes between WT and *OsRUS1-OX* were categorized into one of the three main categories (biological process, cellular component, and molecular function) of the GO classification (Supplementary Table S5). For genes that were upregulated in *OsRUS1-OX*, the top 30 most-enriched GO terms included: 19 terms in the biological process GO category; one term in the cellular component GO category; and ten terms in the molecular function GO category. Of these, three GO terms were significantly enriched: “oxidoreductase activity, acting on the aldehyde or oxo group of donors, disulfide as acceptor” (GO: 0016624, p = 4.5E−02) in the molecular function category; and, “response to water stimulus” (GO: 0009415, p = 4.5E−02) and “response to oxygen-containing compound” (GO: 1901700, p = 4.5E−02) in the biological process category (Fig. 5A). For genes that were downregulated in *OsRUS1-OX*, the top 30 most-enriched GO terms included: 10 GO terms in the biological process GO category; 3 terms in the cellular component GO category; and 17 terms in the molecular function GO category (Fig. 5B).

In organisms, the expression of different genes is typically coordinated so that they may properly execute their biological functions. The roles of the differentially expressed genes in biochemical metabolic pathways and signal transduction pathways can be identified by assessing which pathways show significant enrichment. To identify the biological pathways that may be active in the process of leaf rolling in *OsRUS1-OX*, the differentially expressed genes were mapped to reference pathways in the Kyoto Encyclopedia of Genes and Genomes (KEGG) (http://www.genome.ad.jp/kegg/)^31^. The analysis of significantly enriched pathways was conducted using KOBAS (2.0), where an FDR of ≤ 0.05 indicated a significant enrichment of differentially expressed genes in a pathway. The differentially expressed genes between WT and *OsRUS1-OX* were assigned to 110 KEGG pathways (Supplementary Table S6). The most significantly enriched 20 pathways were selected and displayed in a scatter diagram (Supplementary Figure S7). The classifications indicated that several pathways were significantly differentially regulated between WT and *OsRUS1-OX*, such as carotenoid synthesis (dosa00906), photosynthesis (dosa00195), and plant hormone signal transduction (dosa04075). The pathways with the largest number of differentially expressed genes were the metabolic pathways (dosa01100) and the biosynthesis of secondary metabolites (dosa01110), with 219 and 140 regulated members, respectively. These annotations provided a valuable resource for investigating specific processes, functions and pathways involved in the rapid and dynamic leaf rolling of *OsRUS1-OX*.

### Comparative analysis of metabolic pathways between WT and *OsRUS1-OX*

**Figure 6:**
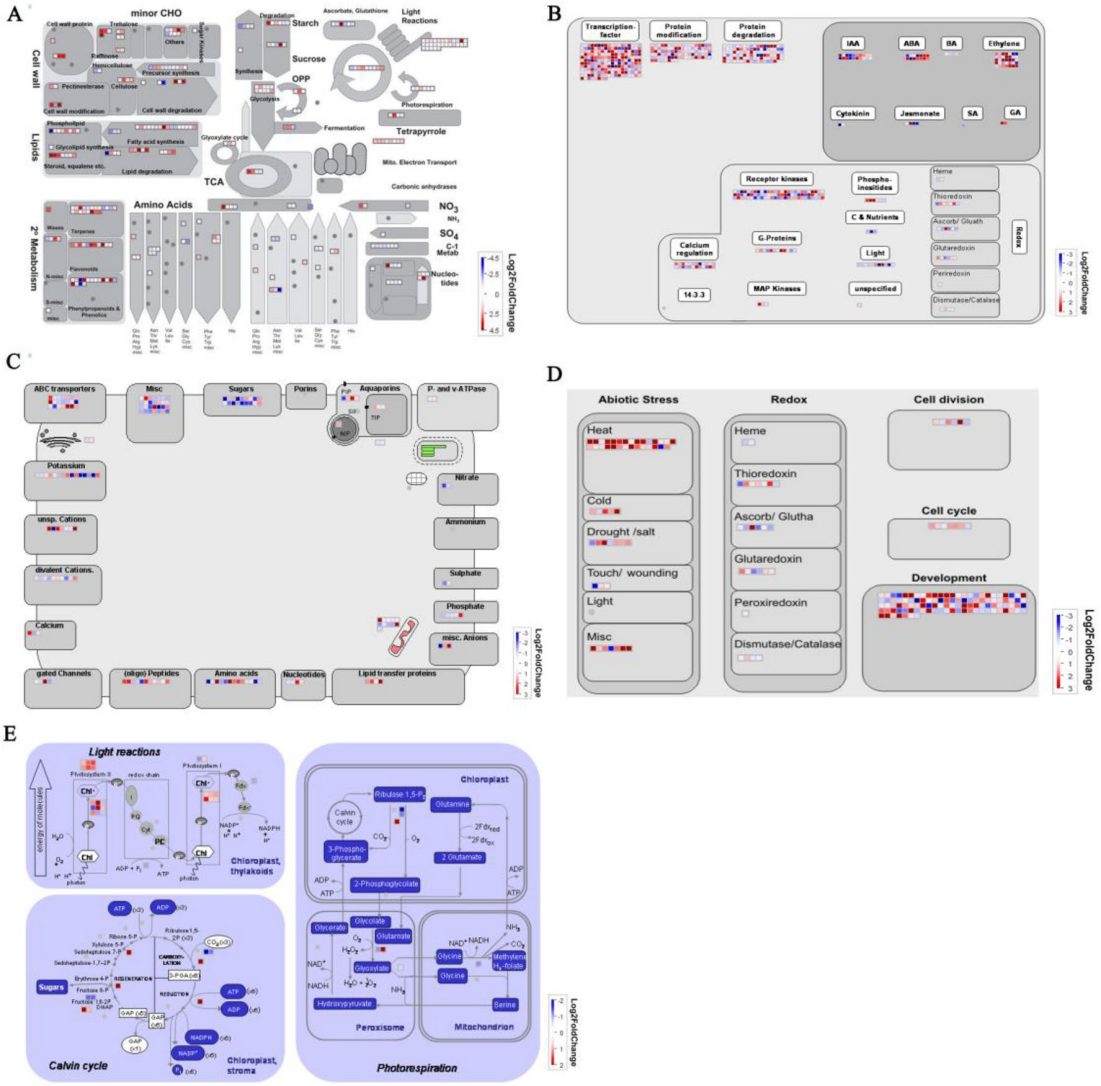
Mapman analysis of differentially expressed genes of WT vs *OsRUS1-OX*. (**A**) metabolism overview; (**B**) regulation overview; (**C**) transport overview; (**D**) cellular response overview; (**E**) photosynthesis. Up- and Down- regulated DEGs are represented with blue and red squares, respectively with log_2_ (Fold Change values).

To understand functional insights of all DEGs, MapMan pathway annotator (version3.6.0RC1) was used for the metabolic and regulatory pathway analysis of DEGs (Supplementary Table S7)^32^. General metabolism analysis displayed that most DEGs in cell wall proteins, cell wall modification, cell wall degradation, cellulose synthesis, C3 cycle, glycolysis pathway, fatty acid synthesis, lipid degradation, raffinose biosynthesis, galactinol biosynthesis were up-regulated in leaves of *OsRUS1-OX.* Results showed that transcripts of ATP synthesis in mitochondrial electron transport were significantly up-regulated in leaves of *OsRUS1-OX* (Fig. 6A, Supplementary Table S8). Metabolism regulations were mainly involved in transcription regulation, postranslational modification, protein degradation, signal receptor kinases and hormone regulation, etc. (Fig. 6B, Supplementary Table S9). The transcripts of biosynthesis and degradation of IAA, ABA and Ethylene were either up or down regulated in leaves of *OsRUS1-OX.* And the transcripts of some receptor like kinases were also significantly up or down regulated in leaves of *OsRUS1-OX* (Fig. 6B, Supplementary Table S9)*.* In photosynthesis pathway analysis, light harvesting chlorophyll a binding protein family and photosystem II subunit protein family genes were significantly up regulated in leaves of *OsRUS1-OX* (Fig. 6E, Supplementary Table S12)*.* These results indicated that plant hormones, light sensing and receptor like kinases played important roles in rapid leaf rolling in *OsRUS1-OX.*

The leaf rolling of *OsRUS1-OX* might also relate to its increased transportation across plasma membrane. According to the transport overview, 21 genes in ABC (ATP BINDING CASSETTE) transporters and multidrug resistance systems with ATPase coupled to transmembrane movement of substances were significantly up-regulated in *OsRUS1-OX* (Fig. 6C, Supplementary Table S10)*.* For example, *Os03g0281900* up-regulated 17.9 times, *PDR11* (*PLEIOTROPIC DRUG RESISTANCE 11*), *PDR12*, *ABCB1* and *WBC11* (*WHITE-BROWN COMPLEX 11*) up-regulated more than two times compared with wildtype. While genes in non-specific cation transport system, divalent cationic transport system, mate efflux family protein, potassium and calcium ion transport system were significantly down-regulated in *OsRUS1-OX.* For example, *Os07g0561800*, *Os11g0637100* down-regulated 8.1 and 6.3 times compared with wildtype, respectively (Fig. 6C, Supplementary Table S10). The substance transportation change across plasma membrane of *OsRUS1-OX* is possibly the cause of rapid leaf rolling phenotype under sunlight.

There were 29 heat stress response genes identified in DEGs. Most of them encoded heat shock proteins, and they were up-regulated in *OsRUS1-OX.* These may explain the rapid response of *OsRUS1-OX* leaves to sunlight. Germin-like protein family genes, which play important roles in abiotic stresses^33^, were also obviously up-regulated in *OsRUS1-OX.* The *LATE EMBRYOGENESIS ABUNDANT* (*LEA*) genes were significantly down-regulated in *OsRUS1-OX* (Fig. 6D, Supplementary Table S11)*.* The leaf rolling/expanding is dependent on the water dehydration/absorption of bulliform cells. The highly expressed *LEA* of *OsRUS1-OX* may cause the more rapid water dehydration/absorption of bulliform cells response to sunlight/shading. Thus, the rapid leaf rolling/expanding phenotype of *OsRUS1-OX* was observed under sunlight/shading.

### The verification of RNA-Seq data by qRT-PCR

**Figure 7:**
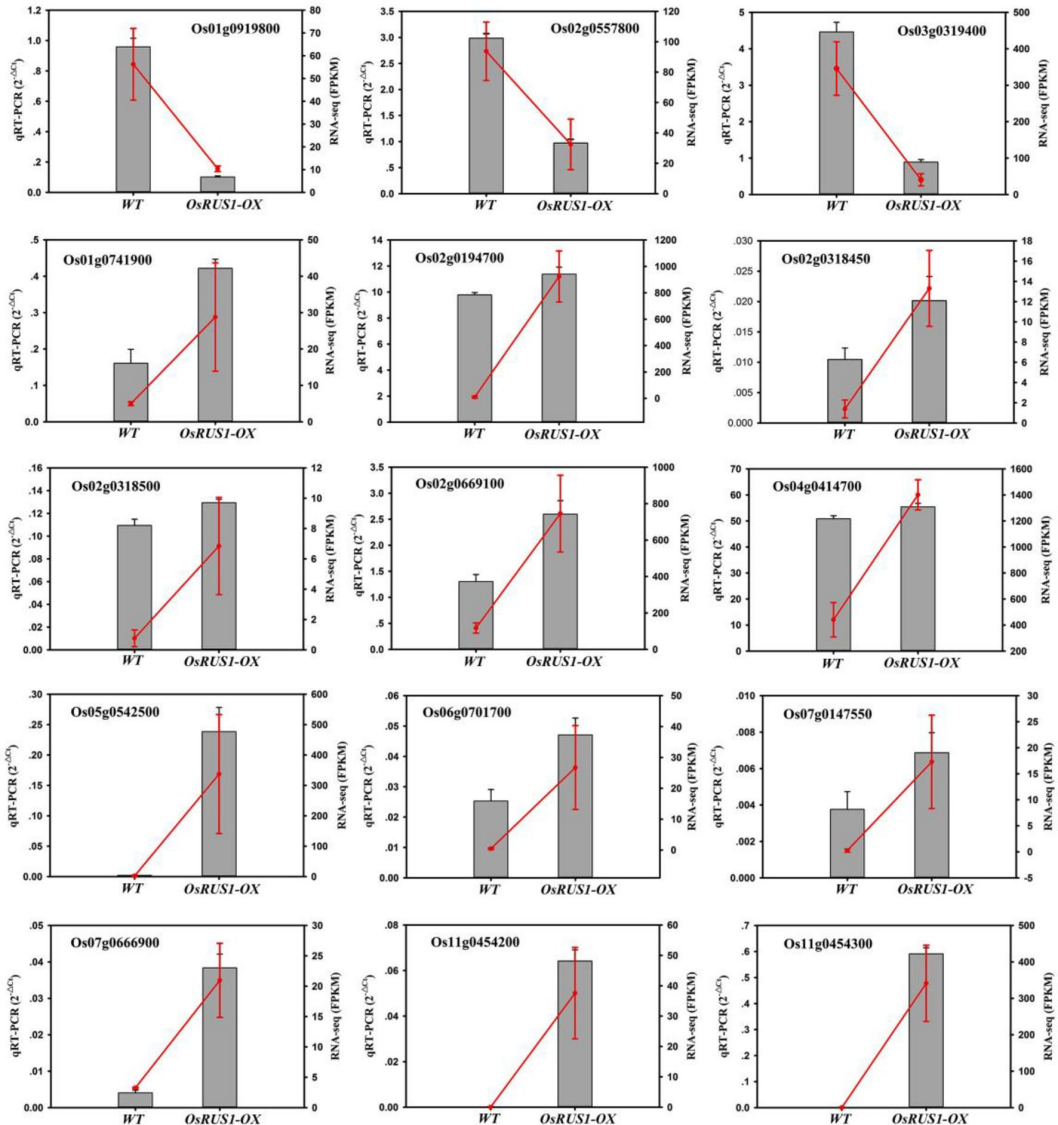
Verification of differentially expressed genes between WT and *OsRUS1-OX* by qRT-PCR. Note: Histograms represent the results of the qRT-PCR assays, using the 2^−ΔCt^ algorithm, with the scale on the left ordinate of each graph. Red dots represent the results of the FPKM analyses, with the scale on the right ordinate of each graph. WT, the wildtype of ZhongHua11; *OsRUS1-OX*, the *OsRUS1-GFP* Overexpression transgenic plant.

In order to verify the differentially expressed genes identified by RNA-seq, qRT-PCR assays for 15 selected genes were performed on independently collected samples that were in the same developmental stage as those used for the RNA-Seq analysis. The 15 genes, *Os02g0669100* (*DEHYDRIN*), *Os05g0542500* (*LEA3*), *Os11g0454300* (*RAB21*) and *Os11g0454200* (*RAB16B*)^34–38^, *Os03g0319400* (*CBL-INTERACTING PROTEIN KINASE 3*, *OsCIPK3*)^39,40^, *Os06g0701700* (*HIGH AFFINITY K*^+^ *TRANSPORTER 1*, *OsHKT1*)^41,42^, *Os07g0666900* (*Na*^+^*/H*^+^ *ANTIPORTER* 1, *OsNHX1*)^43–45^, *Os02g0194700* (*LIPOXYGENASE*)^46^, *Os01g0919800* (*OsPIN5A*)^47,48^, *Os07g0147500* (Photosystem II *PsbR*)^49,50^, *Os04g0414700* (Photosystem I *PsaO*)^51–53^, *Os01g0741900* (*IAA6*)^54^, *Os02g0557800* (Signal transduction response regulator)^55,56^, *Os02g0318450* (ABC transporter-like domain containing protein) and *Os02g0318500* (*OsPDR4*)^57,58^, were chosen because of their potential function in rice leaf rolling. Among the selected 15 differentially expressed genes, 12 genes showed increased expression and 3 genes showed decreased expression in the *OsRUS1-OX*. All of the 15 genes showed the same expression patterns in the qRT-PCR assays as in the RNA-Seq data (Fig. 7). The gene expression patterns between qRT-PCR and RNA-Seq data were well matched, indicating that the RNA-Seq data were credible.

## Discussion

### The rapid and dynamic leaf rolling phenotype of the *OsRUS1-OX* rice transgenic line

Other phenotypes of the *OsRUS1-OX* rice transgenic lines were observed, such as: The length of the flag leaf of *OsRUS1-OX* is not significantly different from that of WT, but its width is significantly narrower than that of WT; The leaf angle of *OsRUS1-OX* is significantly larger than that of WT throughout the developmental period; The seed setting rate of *OsRUS1-OX* is slightly lower than that of WT, but its panicle is significantly longer than that of WT (data not shown), etc. In this study, we focused on the rapid and dynamic leaf rolling phenotype of *OsRUS1-OX*. The leaf rolling phenotype in rice is one of the most important agronomic traits. Moderate leaf rolling is an important part of ideotype for breeding higher productivity rice cultivars. Therefore, finding the leaf rolling phenotype and understanding the mechanisms behind it should be of practical value, and it also has the meaning as basic scientific research. The causes of leaf rolling phenotypes in rice can be divided into rice leaf developmental-gene mutation related and environmental factor induced types. Most of the rice leaf rolling phenotypes reported are due to rice leaf developmental-gene mutations, while only a few rice-leaf rolling phenotypes are caused by environmental factors. We observed that the leaves of *OsRUS1-OX* plants would roll under sunlight, and expand under shading. Additionally, the rolled to expanded, or expanded to rolled, state change of *OsRUS1-OX* line leaves occurred within a few minutes of sunlight condition change (Fig. 1). To the best of our knowledge, no leaf-rolling-related function has been reported in *AtRUS* gene studies, and this rapid and dynamic sunlight induced rice leaf rolling/expanding has not been reported before.

RNA-Seq is becoming a powerful and affordable technology due to the advancement of next generation sequencing. RNA-Seq has been broadly used in many plants, such as sweet cherry^59^, rice^60^, tea^61^, winter rye^62^, and *Plumbago auriculata*^63^, to find differentially expressed genes between samples, and to dissect the mechanisms behind many observed phenotypes. In order to dissect the mechanism behind the leaf rolling phenotype in *OsRUS1-OX* lines, an RNA-Seq approach was used to find differentially expressed genes between WT and *OsRUS1-OX*. In this experiment, 2920 differentially expressed genes were identified, in which 1660 were upregulated and 1260 were downregulated (Supplementary Table S2). These differentially expressed genes belong to various GO terms (Fig. 5), in which GO: 0009415 (response to water stimulus) is the most likely class of genes involved in the leaf rolling/expanding phenotype of *OsRUS1-OX*. Furthermore, these differentially expressed genes can be assigned to 110 KEGG pathways (Supplementary Table S5). 42 of the 222 genes in the KEGG pathway dosa04075 (Plant hormone signal transduction) were detected as differentially expressed between WT and *OsRUS1-OX* (Supplementary Table S6). Plant hormone signal transduction and dehydration of bulliform cells are the causes of plant leaf rolling^64–66^. Although not all the differentially expressed genes are caused by leaf rolling phenotype of *OsRUS1-OX*, we postulated that the molecular basis of the leaf rolling of *OsRUS1-OX* was concealed in these groups of identified differentially expressed genes. Therefore, these differentially expressed genes in GO: 0009415 and dosa04075 will be key targets in our next experiments to dissect the mechanism behind this phenotype.

### The possible mechanism of rapid and dynamic leaf rolling in the *OsRUS1-OX*

The expressions of reported leaf rolling genes, such as *OsSRL1*^5^, Os*SRL2*^5^, *OsSLL1*^11^, *OsSLL2*^67^, *OsADL1* (*ADAXIALIZED LEAF1*)^68^, *OsAGO7* (*ARGONAUTES 7*)^69^, *OsACL1* (*ABAXIALLY CURLED LEAF 1*)^70^, *RL14*^71^, were data-mined in our RNA-Seq data. Our results showed that none of these genes was differentially expressed between WT and *OsRUS1-OX*, which indicated that the dynamic leaf rolling of *OsRUS1-OX* was not caused by abnormal leaf development due to changes in the expression of these genes.

According to our RNA-Seq data, the expressions of six genes of the KEGG pathway "Photosynthesis—antenna proteins" were significantly increased in *OsRUS1-OX* (Supplementary Table S6), which may be the reason why *OsRUS1-OX* was more sensitive to sunlight than WT. Carotenoids are a signaling molecule precursor in the response to external stimulation of a plant^72^, and 17 genes of the KEGG pathway "Carotenoid biosynthesis" were determined to be significantly differentially expressed between WT and *OsRUS1-OX* (Supplementary Table S6). Plant hormones are reported to be involved in the response to environmental stimuli, and 42 genes of the KEGG pathway "Plant hormone signal transduction" were differentially expressed between WT and *OsRUS1-OX* (Supplementary Table S6). Ion and water transport across bulliform cells is involved in rice leaf rolling^73^. 255 genes for “transporters” (GO: 0005215) and “antiporters” (GO: 0015297) were differentially expressed between WT and *OsRUS1-OX* (Supplementary Table S5). This indicated that the transport of ions, water and other substances were likely significantly changed between WT and *OsRUS1-OX.* The expressions of six genes of the "response to water stimulus" category (GO: 0009415) (Supplementary Table S5), and some dehydrin genes, such as *Os05g0542500* (*LEA3*), *Os11g0454300* (*RAB21*), *Os11g0454200* (*RAB16B*) and *Os02g0669100* (Supplementary Table S3), were significantly increased in *OsRUS1-OX*. This indicated that *OsRUS1-OX* plants were under physiological dehydration, and that the leaf rolling of *OsRUS1-OX* occurred in response to this physiological dehydration^74^.

**Figure 8:**
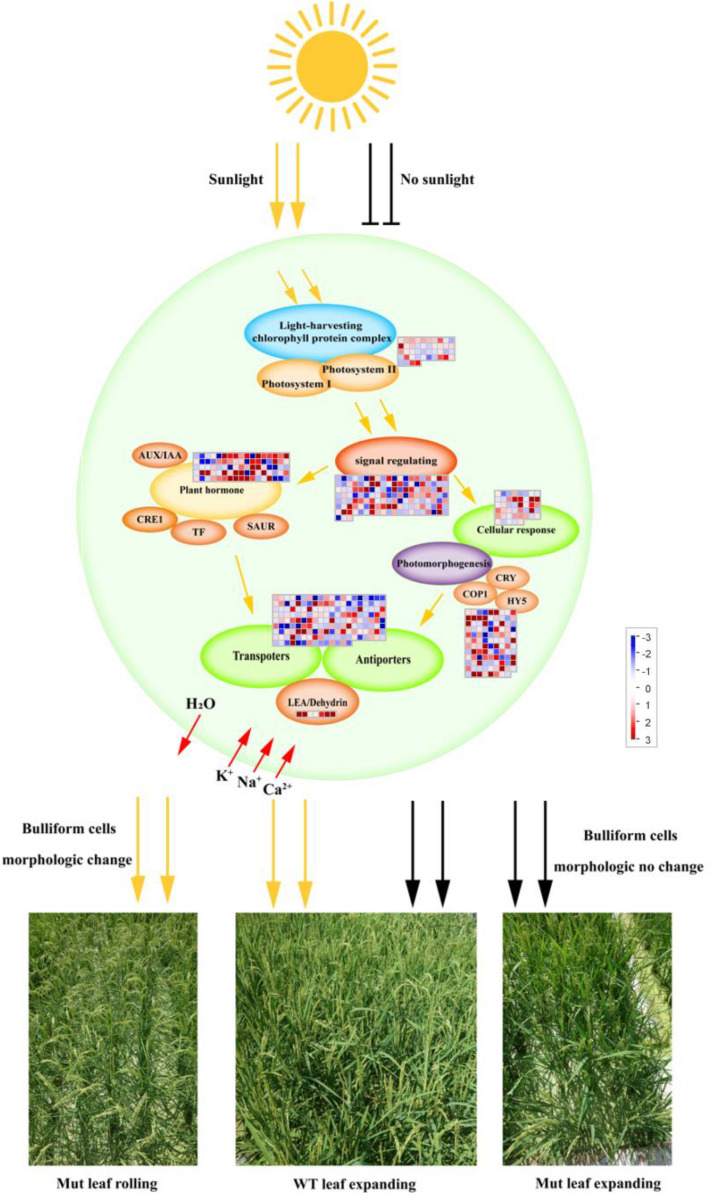
A working model explaining the rapid and dynamic leaf rolling in the *OsRUS1-OX* in response to sunlight. Note: The expressions of some genes in mesophyll cells (green cycle) are regulated due to the overexpression of *OsRUS1-GFP*. The transgenic plants become more sensitive to sunlight. The sunlight will trigger the pathway to cause the leaf rolling of *OsRUS1-OX*. The WT is not as sensitive to sunlight as *OsRUS1-OX*, and the pathway will not be triggered under the same conditions. If the sunlight is shaded, although the pathway is still there, it is not activated, and the bulliform cells of *OsRUS1-OX* will be refilled with water to cause leaf expansion.

According to our analyses, a possible mechanism for the rapid and dynamic leaf rolling of *OsRUS1-OX* is proposed (Fig. 8). In our model, the overexpression of *OsRUS1-GFP* induces high expression of sunlight sensing genes, making the transgenic lines very sensitive to sunlight. Sunlight will gradually trigger plant hormone signal transduction, leading to activation of transporters, antiporters and dehydrins. Therefore, the leaves of *OsRUS1-OX* will sense physiological dehydration, which will cause the bulliform cells to lose water, quickly leading to leaf rolling. If the sunlight is shaded, the above pathway is still present, but there will be no sunlight to trigger it. No physiological dehydration will be detected by plant, and the bulliform cells will refill with water leading to leaf expansion.

In summary, a rapid and dynamic leaf rolling phenotype in response to sunlight is reported for the *OsRUS1-OX*. In this study, an RNA-Seq approach was used to detect transcriptional differences between WT and *OsRUS1-OX*. A total of 2920 differentially expressed genes were found, in which 1660 genes were significantly upregulated and 1260 genes were significantly downregulated in the *OsRUS1-OX*. The reliability of RNA-Seq data was verified by qRT-PCR analyses on 15 selected genes. A possible mechanism for the rapid and dynamic leaf rolling of *OsRUS1-OX* was proposed based on deep analysis of our RNA-Seq data. The differentially expressed genes in GO: 0009415 (response to water stimulus) and KEGG pathway dosa04075 (Plant hormone signal transduction) will be our key targets to further explore the mechanism behind this phenotype in future studies.

## Materials and methods

### Plant materials

Rice plants were cultivated in an isolated experimental field under natural growth conditions. Field management adhered to normal agricultural practices. When rice plants were at the heading stage, the flag leaves were collected on a sunny summer day (11:00 am, WT with expanded leaves, *OsRUS1-OX* with rolled leaves). Leaves from three WT and three *OsRUS1-OX* line plants were pooled as two separate samples. Leaves from WT and *OsRUS1-OX* were independently collected three times to create three biological replicates. All samples were frozen in liquid nitrogen immediately, and stored at − 80 °C until use. The samples were sent to Novogene (Beijing, China) for transcriptome sequencing. We declare that all genetically modified rice cultivation and treatment were carried out in accordance with the regulations of the Ministry of Agriculture of China and are supervised by the Science and Technology Department of South China Agricultural University.

### RNA extraction and quality test

RNA was isolated from WT and *OsRUS1-OX* flag leaves using the TIANGEN RNAprep Pure Plant Kit (Tiangen, Beijing, China). The quality of isolated RNA was checked using 1% agarose gel, and the RNA purity was detected using the NanoPhotometer® spectrophotometer (IMPLEN, CA, USA). The RNA concentration was measured using the Qubit® RNA Assay Kit with the Qubit® 2.0 Fluorometer (Life Technologies, CA, USA). The RNA integrity was evaluated using the RNA Nano 6000 Assay Kit of the Bioanalyzer 2100 system (Agilent Technologies, CA, USA). The qualified RNA was used for following RNA-Seq library construction.

### RNA-Seq library construction and sequencing

In this experiment, NEBNext® Ultra™ RNA Library Prep Kit for Illumina® (NEB, USA) was used for library construction. Briefly, 3 μg of total RNA per sample was used as starting material. Through poly-T oligo-attached magnetic beads, mRNA was isolated from total RNA, and fragmented using divalent cations under elevated temperature in 5X NEBNext first strand synthesis reaction buffer. First strand cDNA synthesis was carried out using random hexamer primers and M-MLV reverse transcriptase (RNase H^-^), and second strand cDNA was synthesized subsequently using DNA polymerase I and RNase H. Remaining overhangs were blunted via exonuclease/polymerase activities. NEBNext adaptors with hairpin loop structure were then ligated to cDNA fragments with 3′ ends adenylated. In order to select 150–200 bp length cDNA fragments, the cDNA fragments were purified with AMPure XP system (Beckman Coulter, Beverly, USA). Then, 3 μl USER Enzyme (NEB, USA) was used with 150–200 bp length adaptor-ligated cDNA fragments at 37 °C for 15 min followed by 5 min at 95 °C. PCR was performed using Phusion High-Fidelity DNA polymerase, Universal PCR primers and Index (X) Primer. PCR products were purified using AMPure XP system and library quality was evaluated on the Agilent Bioanalyzer 2100 system. Following the manufacturer’s instructions, the index-coded samples were clustered on a cBot Cluster Generation System using TruSeq PE Cluster Kit v3-cBot-HS (Illumia). After clustering, the cDNA library products were sequenced on an Illumina Hiseq platform and 125 bp/150 bp paired-end raw reads were generated.

### Quality control

Raw data (reads) in fastq format were filtered through in-house perl scripts. After adapter, reads containing ploy-N, and low quality reads were removed from the raw data, clean data (reads) were generated. The Q20, Q30 and GC content of the clean data were calculated thereby. All of the following analyses were based on the high quality clean data.

### Reads mapping to the reference genome

From the public available genome website (ftp://ftp.ensemblgenomes.org/pub/release-23/plants/fasta/oryza_sativa/dna/), the rice reference genome and gene model annotation files were downloaded. Index of the reference genome was built using Bowtie v2.2.3 and paired-end clean reads were aligned to the reference genome using TopHat v2.0.12^75^. TopHat was selected as the mapping tool due to TopHat can generate a database of splice junctions based on the gene model annotation files, and thus a better mapping result will be generated than other non-splice mapping tools.

### Quantification of gene expression level

We used HTSeq v0.6.1 to count the read numbers mapped to each rice gene^76^. And FPKM (expected number of Fragments Per Kilobase of transcript sequence per Millions base pairs sequenced) of each rice gene was calculated based on the length of the gene and read counts mapped to this gene. Due to FPKM takes both the effects of sequencing depth and gene length on the read counts into consideration, thus it is currently the most commonly used method for reckoning gene expression levels^28^.

### Differential expression analysis

The differential expression analysis between WT and *OsRUS1-OX* was carried out using the DESeq R package (1.18.0)^77^. By using a model based on the negative binomial distribution, DESeq R package offers statistical routines for finding out differential expression in digital gene expression data. The resulting P-values were corrected using Benjamini and Hochberg’s approach for managing the false discovery rate (FDR). Gene with adjusted P-value less than 0.05 determined by DESeq R package was considered as differentially expressed.

### GO, KEGG and MapMan analysis of differentially expressed genes

Gene Ontology (GO) enrichment analysis of identified differentially expressed genes was performed by the GOseq R package^30^, where gene length bias was adjusted. GO terms of differentially expressed genes with corrected P-value less than 0.05 were assigned significantly enriched.

KEGG (http://www.genome.jp/kegg/) is a database resource for understanding high-level functions and utilities of the biological pathway, therefore it is good to understand gene’s function^31^. We used KOBAS software to identify the statistical enrichment of differentially expressed genes in KEGG pathways^78^.

MapMan pathway annotator (version 3.6.0 RC1, https://mapman.gabipd.org/mapman/) was used to display the graphical overview of metabolism pathways^32^. Release Genome of Osa_RAPDB_v1 was used as mapping reference data to group and display. IDs of rice genes and their log2FC values were imported to MapMan as an experimental data set. The Wilcoxon rank-sum test with Benjamini Hochberg corrected was used to analyze which bins/pathways were differentially enriched between two samples.

### Validation of RNA-seq data by quantitative RT-PCR assays

Validation of RNA-seq data for 15 phenotype-related differentially expressed genes was performed by qRT-PCR. The primers of selected genes were designed by using Primer premier 5 software (PREMIER Biosoft, Palo Alto, CA, USA) and synthesized by IGE Biotechnology Co., LTD (Guangzhou, China). cDNAs were synthesized from 1 μg of total RNA using reverse transcriptase M-MLV (RNase H^-^) (Takara, Dalian, China). qRT-PCR reactions were performed using the Biotool™ 2 × SYBR Green QPCR Master Mix (Biotool, Shanghai, China) on a CFX96 real-time system (Bio-Rad, CA, USA) following the manufacturer’s instructions. The housekeeping gene *OsACTIN1* (Os03g0718100) was used to normalize the expression level of genes in our experiment^79,80^. Each plate was repeated three times in independent runs for all reference and selected genes. Gene expression was evaluated by the 2^−ΔCt^ method^81^. The qRT-PCR primers used are listed in Supplementary Table S1.

## Supplementary Information

Supplementary Figure S1.

Supplementary Figure S2.

Supplementary Figure S3.

Supplementary Figure S4.

Supplementary Figure S5.

Supplementary Figure S6.

Supplementary Figure S7.

Supplementary Table S1.

Supplementary Table S2.

Supplementary Table S3.

Supplementary Table S4.

Supplementary Table S5.

Supplementary Table S6.

Supplementary Table S7.

Supplementary Table S8.

Supplementary Table S9.

Supplementary Table S10.

Supplementary Table S11.

Supplementary Table S12.

## Data Availability

The RNA-Seq data generated in this experiment were deposited in GEO database of NCBI (https://www.ncbi.nlm.nih.gov/geo/), and the accession number is GSE128886.

## Abbreviations

DGE: Digital gene expression
ENCODE: Encyclopedia of DNA Element
FDR: False discovery rate
FPKM: Expected number of Fragments Per Kilobase of transcript per Millions base pairs sequenced
GO: Gene otology
IAA: Indole-3 acetic acid
KEGG: The Kyoto Encyclopedia of Genes and Genomes
Q30: The percentage of bases for which the Phred value was larger than 30
*OsRUS1-OX*: *OsRUS1-GFP* Overexpression transgenic rice line
*RUS*: *ROOT UVB SENSITIVE*
VB6: Vitamin B6
VLF: Very-low fluence
*WXR*: *WEAK AUXIN RESPONSE*
