# Supplementary material for: Transcriptomic analysis of OsRUS1 overexpression rice lines with rapid and dynamic leaf rolling morphology
Source: Sci Rep. 2022 Apr 25;12:6736. doi: 10.1038/s41598-022-10784-x (PMC9038715; doi:10.1038/s41598-022-10784-x)
Supplement: Supplementary file 1 — Supplementary Figure S1. [file 41598_2022_10784_MOESM1_ESM.docx]

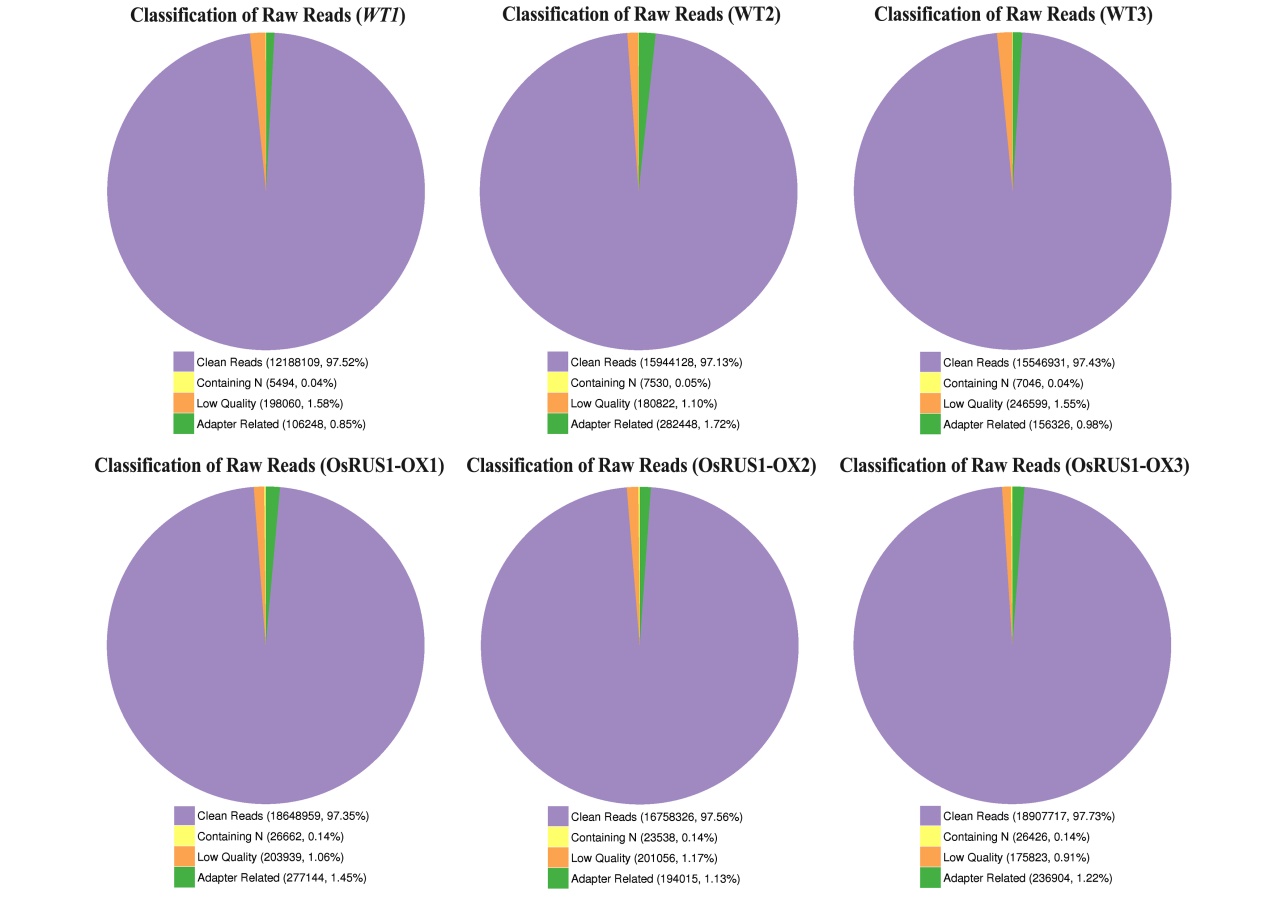


**Supplementary Figure S1. The classification of WT and *OsRUS1-OX* RNA-Seq raw reads**

The original reads of RNA-Seq data are composed of four kinds of sequences: cleaning reads, containing N, low quality and adapter related sequences. The numbers in parentheses represent the total reads of that category and its percentage of total reads.
