# Supplementary material for: Transcriptomic analysis of OsRUS1 overexpression rice lines with rapid and dynamic leaf rolling morphology
Source: Sci Rep. 2022 Apr 25;12:6736. doi: 10.1038/s41598-022-10784-x (PMC9038715; doi:10.1038/s41598-022-10784-x)
Supplement: Supplementary file 2 — Supplementary Figure S2. [file 41598_2022_10784_MOESM2_ESM.docx]

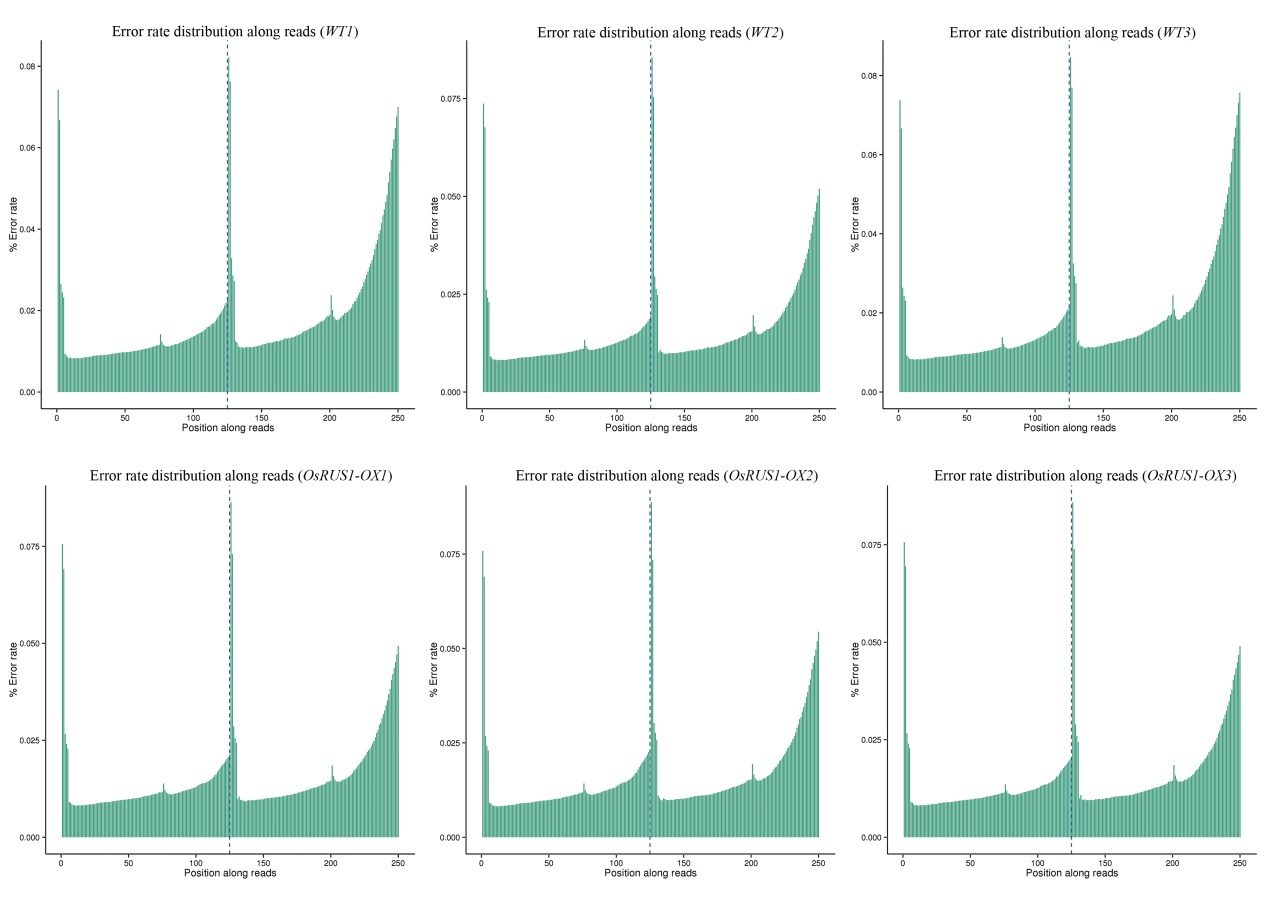


**Supplementary Figure S2. The error rate distribution along WT and *OsRUS1-OX* RNA-Seq reads**

The sequencing error rate is related to the base quality of reads, which is influenced by many factors, such as the sequencing instrument, sequencing reagents and sequencing samples. For RNA-Seq, the distribution of the sequencing error rate has two traits: The sequencing error rate increases with the length of reads; and, the first six bases of each read generally have a higher rate of sequencing errors due to the random primers used in RNA-Seq library construction. As a general rule, the sequencing error rate of each base should be less than 0.5%. In this figure, the Abscissa is the base position of reads; the Ordinate is the single base error rate of reads.
