# Supplementary material for: Transcriptomic analysis of OsRUS1 overexpression rice lines with rapid and dynamic leaf rolling morphology
Source: Sci Rep. 2022 Apr 25;12:6736. doi: 10.1038/s41598-022-10784-x (PMC9038715; doi:10.1038/s41598-022-10784-x)
Supplement: Supplementary file 3 — Supplementary Figure S3. [file 41598_2022_10784_MOESM3_ESM.docx]

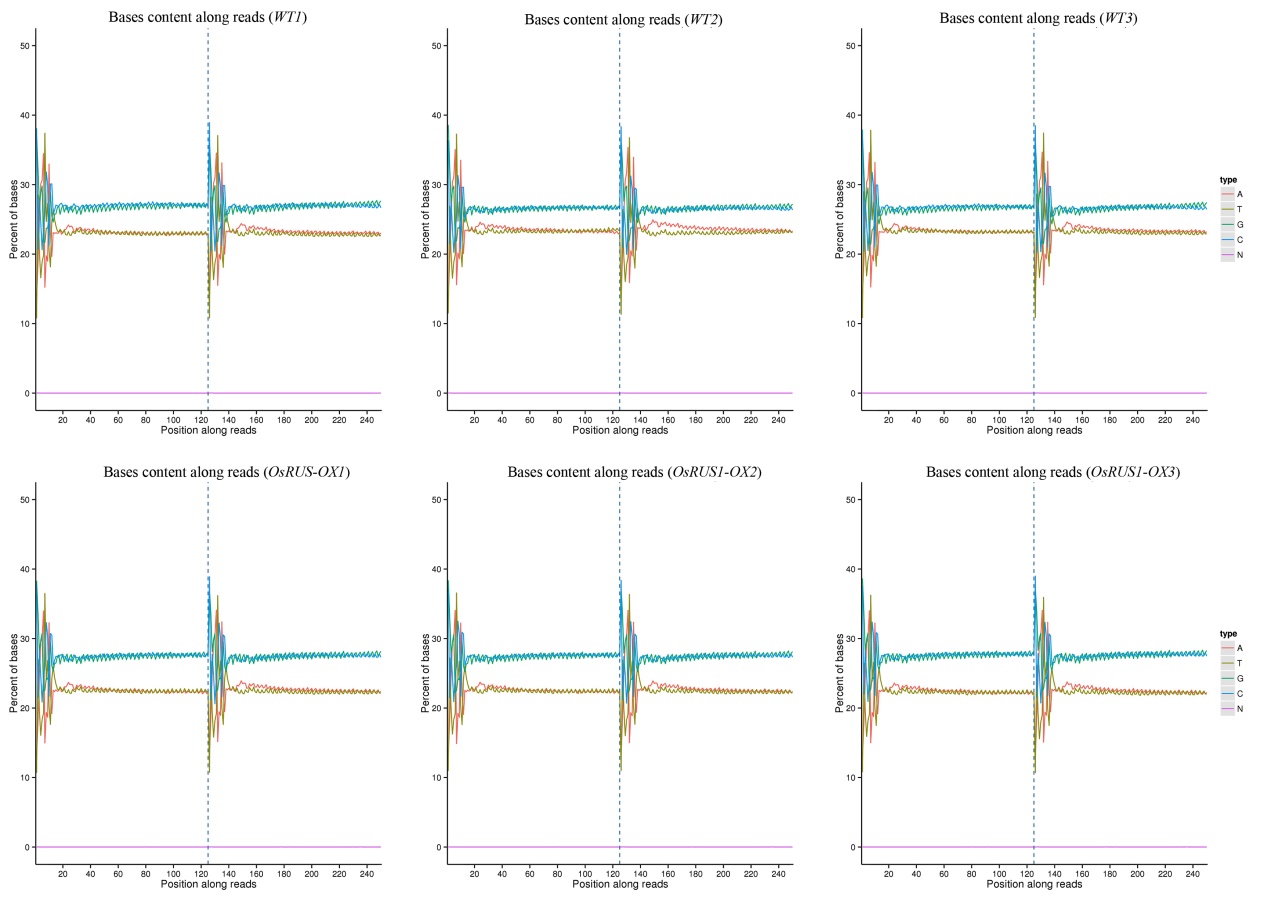


**Supplementary Figure S3. The GC content distribution along WT and *OsRUS1-OX* RNA-Seq reads**

In this figure, the Abscissa is the base position of reads; the Ordinate is the percentage of each base. Different colors represent different base types.
