# Supplementary material for: Transcriptomic analysis of OsRUS1 overexpression rice lines with rapid and dynamic leaf rolling morphology
Source: Sci Rep. 2022 Apr 25;12:6736. doi: 10.1038/s41598-022-10784-x (PMC9038715; doi:10.1038/s41598-022-10784-x)
Supplement: Supplementary file 4 — Supplementary Figure S4. [file 41598_2022_10784_MOESM4_ESM.docx]

**
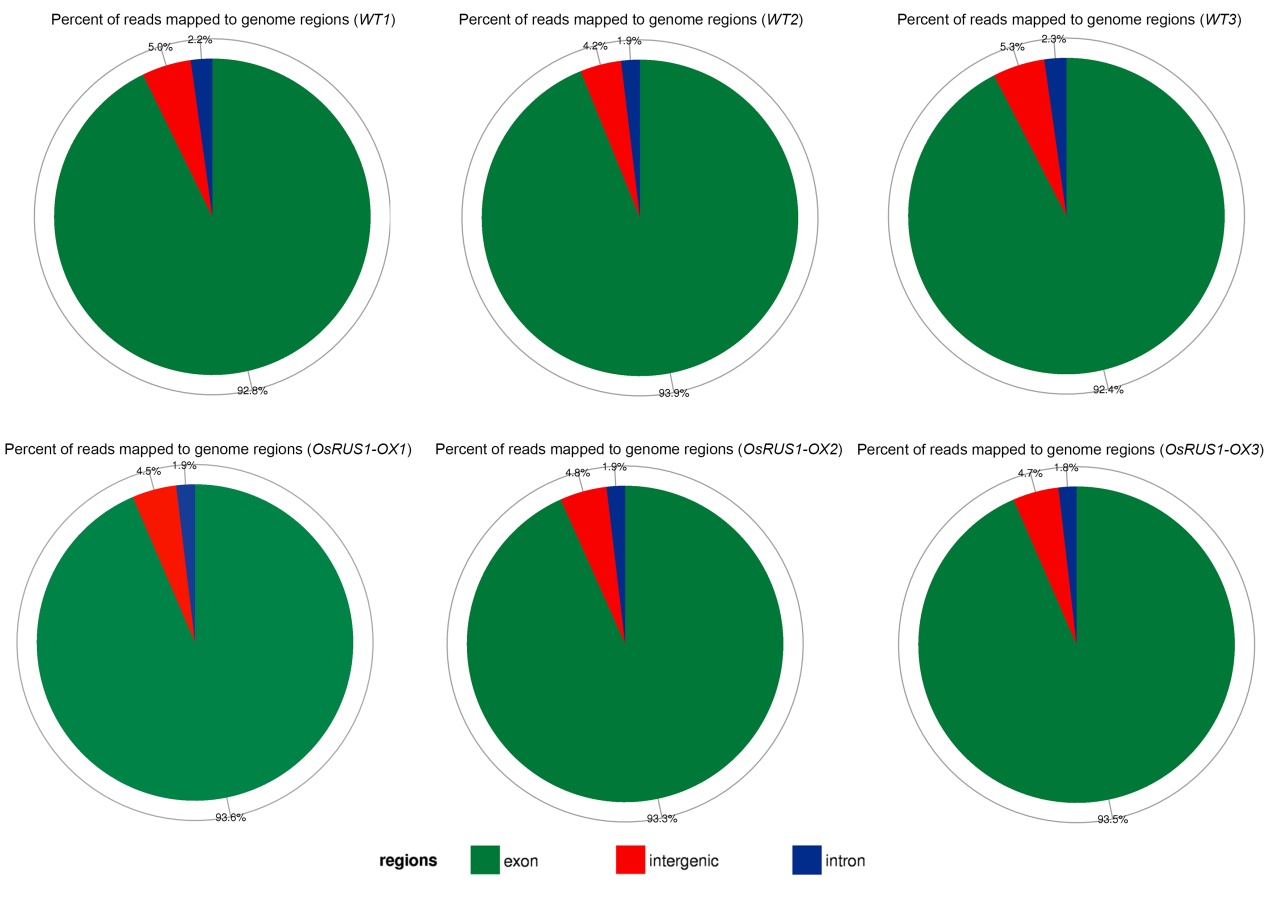
**

**Supplementary Figure S4. The percentage of WT and *OsRUS1-OX* RNA-Seq reads mapped to rice genome regions**

In this figure, the percentage of clean reads mapped to exon, intron and intergenic regions of rice genome is displayed.
