# Supplementary material for: Transcriptomic analysis of OsRUS1 overexpression rice lines with rapid and dynamic leaf rolling morphology
Source: Sci Rep. 2022 Apr 25;12:6736. doi: 10.1038/s41598-022-10784-x (PMC9038715; doi:10.1038/s41598-022-10784-x)
Supplement: Supplementary file 5 — Supplementary Figure S5. [file 41598_2022_10784_MOESM5_ESM.docx]

**
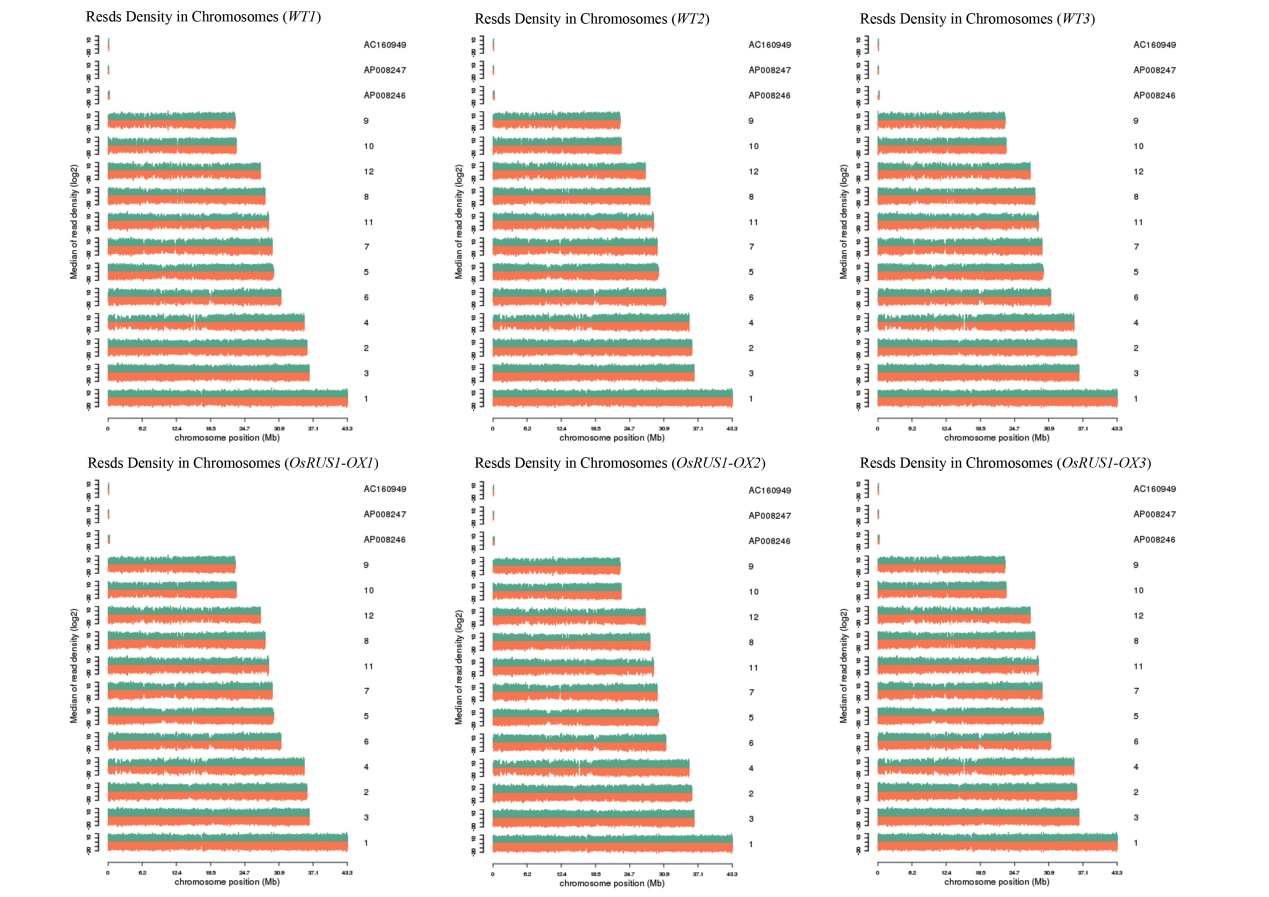
**

**Supplementary Figure S5. The distribution and density of WT and *OsRUS1-OX* total mapped reads on rice chromosomes**

In this distribution and density of reads on the rice genome plot, the Abscissa is the length of rice chromosomes (with million bases as the unit); the Ordinate is the log_2_(the mid-value of reads density).
