# Supplementary material for: Transcriptomic analysis of OsRUS1 overexpression rice lines with rapid and dynamic leaf rolling morphology
Source: Sci Rep. 2022 Apr 25;12:6736. doi: 10.1038/s41598-022-10784-x (PMC9038715; doi:10.1038/s41598-022-10784-x)
Supplement: Supplementary file 6 — Supplementary Figure S6. [file 41598_2022_10784_MOESM6_ESM.docx]

**
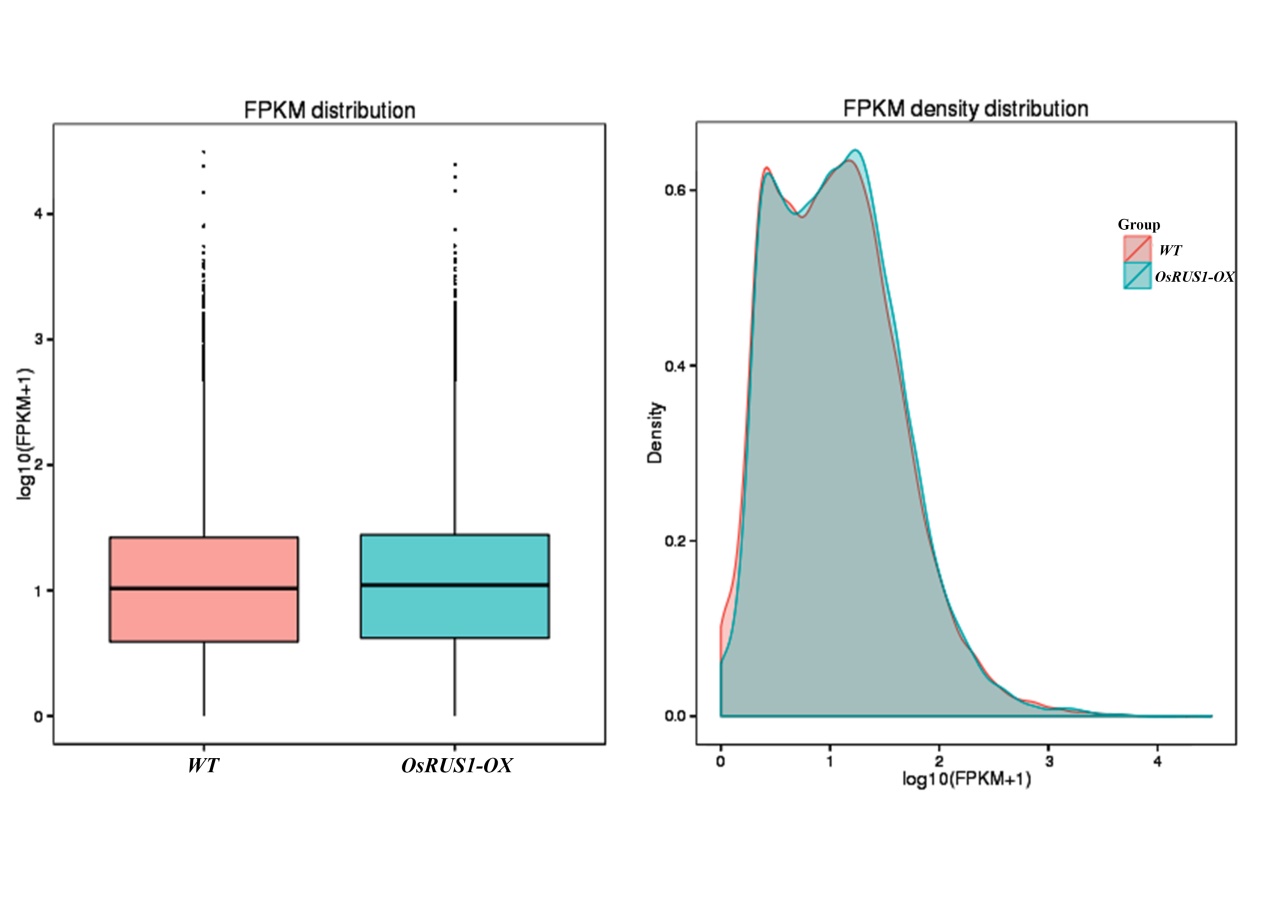
Supplementary Figure S6. Comparison of gene expression levels between WT and *OsRUS1-OX***

In the FPKM distribution figure, the Abscissa is the name of samples; the Ordinate is the value of log_10_(FPKM+1). Each area of the box plot corresponds to five expression levels. From top to bottom, they are the maximum value, upper quartile, mid-value, lower quartile and the minimum value, respectively. In the FPKM density distribution plot, the Abscissa is the value of log_10_(FPKM+1); the Ordinate is the density of differentially expressed genes between WT and *OsRUS1-OX*.
