# Supplementary material for: Transcriptomic analysis of OsRUS1 overexpression rice lines with rapid and dynamic leaf rolling morphology
Source: Sci Rep. 2022 Apr 25;12:6736. doi: 10.1038/s41598-022-10784-x (PMC9038715; doi:10.1038/s41598-022-10784-x)
Supplement: Supplementary file 7 — Supplementary Figure S7. [file 41598_2022_10784_MOESM7_ESM.docx]

**
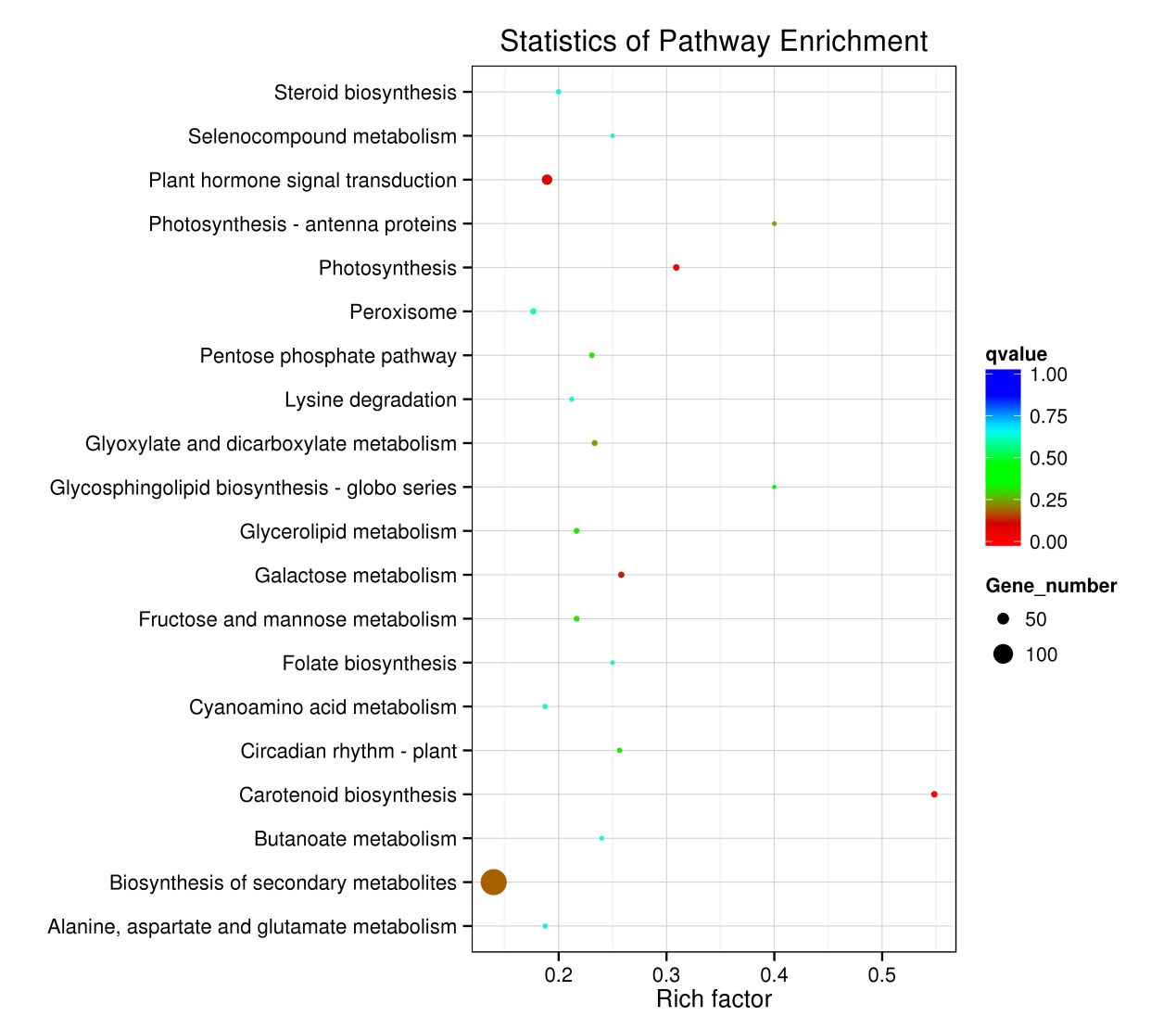
**

**Supplementary** **Figure S7. Scatter plot of differential expressed genes between WT and *OsRUS1-OX* in KEGG pathway enrichment**

In this figure, the 20 most-significantly enriched KEGG pathways are displayed. The Ordinate shows the pathway name; the Abscissa is the rich factor of the KEGG pathway. The dot size indicates the number of differentially expressed genes in the pathway, while the color of the dots corresponds to qvalue ranges.
