# Supplementary material for: Transcriptomic analysis of OsRUS1 overexpression rice lines with rapid and dynamic leaf rolling morphology
Source: Sci Rep. 2022 Apr 25;12:6736. doi: 10.1038/s41598-022-10784-x (PMC9038715; doi:10.1038/s41598-022-10784-x)
Supplement: Supplementary file 8 — Supplementary Table S1. [file 41598_2022_10784_MOESM8_ESM.docx]

**Supplementary Table S1. The primer list of qRT-PCR of selected 15 genes for RNA-Seq data verification**

| **Gene ID** | **Annotation** | **Primer sequence (5'-3')** |
| --- | --- | --- |
| Os01g0741900 | Auxin-responsive protein，  Drought tolerance，  Control of tiller outgrowth | F: AGGAGACAGGATGCTGGTTG  R: AGCAGTAGCTCCAATCAGGG |
| Os02g0557800 | A-type response regulator，  Cytokinin signaling | F: GAGACGATGACCGGTGCTG  R: TGAGCTGGAAGTAGTGCGAC |
| Os04g0414700 | Photosystem I PsaO domain containing protein | F: TTAGGAGGGACCTGAACGTGA  R: CCAGGAACAGCCCAAGGTG |
| Os07g0147550 | Similar to Photosystem II 10 kDa polypeptide | F: GGAAGGCCAATCAAGGGGAA  R: AAAGAGTGACTGCCCAGAGC |
| Os01g0919800 | Similar to Efflux carrier of polar auxin transport | F: ACACTTACGCCAGCTTCGTC  R: ATTCCTGTGCCTGACTTGGA |
| Os02g0194700 | Similar to Lipoxygenase 2.3, chloroplast precursor | F: GCGAGATAGGTTCTCGTGGT  R: TGCTCACGATGGGAAACTCC |
| Os03g0319400 | Serine/threonine protein kinase, Cold stress tolerance,  Abiotic stresses | F: CAAGTGAAGGCTGACGGTTT  R: AGGATTACCCCACAAGACCAG |
| Os06g0701700 | Ion transporter , Na+/K+ symport；Similar to Cation transporter HKT1. | F: TCATCGCCCCTGCTGTTCTA  R: AGACTGAGATGCAGGCAAGC |
| Os07g0666900 | Vacuolar Na+/H+ antiporter,  Salt tolerance；Similar to Sodium/hydrogen exchanger. | F: AAATGGGAGTTTGCCAGTGA  R: TCGGTGCCTTCTTTGTTAGG |
| Os11g0454300 | Similar to Water-stress inducible protein RAB21. | F: CGGCCAGTTCCAGCCGAT  R: CTCCCTCCCATTCCATCA |
| Os11g0454200 | Dehydrin RAB 16B. | F: CGATGGGGATGGGAGGTC  R: GCTGCTGGTGGTTGTTGC |
| Os05g0542500 | Similar to Isoform 2 of Late embryogenesis abundant protein, group 3. LEA-like protein. | F: CACCAAGGACTCTGCCAT  R: CCCCAGCGTGCTCATCAC |
| Os02g0669100 | Dehydrin family protein. | F: GCCTCTTCGACAACCTC  R: CTTCCTCTTGACCACCTC |
| Os02g0318450 | ABC transporter-like domain containing protein. | F: AAATACTGTCAACACTGG  R: TAATACTTGGAACACCTA |
| Os02g0318500 | ABC transporter-like domain containing protein. | F: TTGGGCTGGACATTTGCG  R: TCTCTGGCGGTGGTTGGA |
| Os03g0718100 | ATP binding. ACTIN1 | F: CTTCATAGGAATGGAAGCTGCGGGTA  R: CGACCACCTTGATCTTCATGCTGCTA |

Note: The gene ID in this table is the accession number from RAP-DP rice database; F is the forward primer; R is the reverse primer.
